# Toothpicking and Periodontal Disease in a Neanderthal Specimen from Cova Foradà Site (Valencia, Spain)

**DOI:** 10.1371/journal.pone.0076852

**Published:** 2013-10-16

**Authors:** Marina Lozano, Maria Eulàlia Subirà, José Aparicio, Carlos Lorenzo, Gala Gómez-Merino

**Affiliations:** 1 IPHES, Institut Català de Paleoecologia Humana i Evolució Social, Tarragona, Spain; 2 Àrea de Prehistòria, Universitat Rovira i Virgili (URV).Tarragona, Spain; 3 Unitat d’Antropologia Biològica (GRAPAC), Facultat de Biociències, Universitat Autònoma de Barcelona (UAB), Barcelona, Spain; 4 MINOA Arqueologia i Serveis S.L. I+D, Bellaterra, Spain; 5 Secció d’Estudis Arqueològics Valencians, Diputación Provincial de Valencia, Valencia, Spain; Universidad Europea de Madrid, Spain

## Abstract

We present a Neanderthal maxilla (CF-1) from Cova Foradà site (Oliva, Valencia, Spain) with periodontal disease and evidence of attempts to alleviate pain with the use of a toothpick. Two interproximal grooves have been found on the distal surfaces of the upper left Pm^3^ and M^1^ of CF-1 maxilla. The location, morphology and size of the grooves coincide with other interproximal grooves found on the teeth of other fossil specimens. Heavy dental wear and periodontal disease would have caused the Cova Foradà Neanderthal specimen pain and discomfort, which the individual attempted to mitigate using some kind of dental probe.

## Introduction

A toothpick is a small stick normally made of wood, but also of bamboo, metal, bone or other material with one or two sharp ends to insert between the teeth. Although not recommended by dentists, toothpicks are widely used to remove trapped food particles that irritate or hurt the gums. Although these are modern uses, they very likely have a very long history.

The use of toothpicks is widespread in every culture, and spans from the beginning of the genus *Homo* to modern times. In human evolution, this habit has often been documented in different *Homo* species, from *Homo habilis* 1.84 m.a. ago to modern humans living today [1], [2], [3], [4]. Interproximal grooves on Neanderthal teeth are a common feature, indicating that the habit of picking the teeth with a tool was well established in this species. Agger and colleagues [5] suggest that the use of toothpicks may even constitute evidence of the biological capacity for language.

The aim of this study is to show the correlation between the use of toothpicks and an attempt to alleviate sore gums in a Neanderthal maxilla recovered from the archaeological site of Cova Foradà (Oliva, Valencia, Spain).

**Figure 1:**
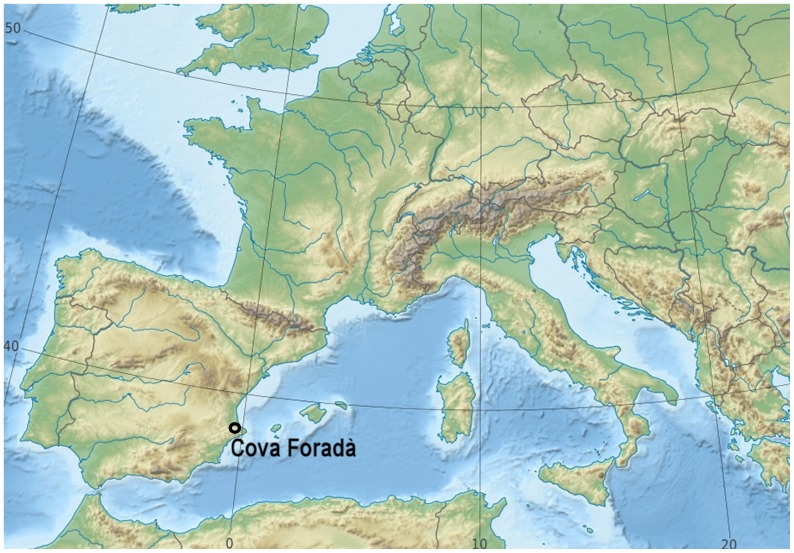
Location of Cova Foradà site.

The Cova Foradà is a cave located in the middle of the central Mediterranean coast on the Iberian Peninsula (Oliva, Valencia) (Fig. 1). The cave presents human occupation from the Mousterian to the Mesolithic and also Bronze Age, Iberian and medieval period. The first archaeological levels are dated in the Bronze Age: 5,633±31 BP (6.437 cal B.P.) (charcoal layer 1, CSIC-1492) and 6196±34 BP (7.220 cal B.P.) (charcoal layer 2, CSIC-1493). The layer 11 had faunal remains dated at Upper Paleolithic 16,960+100 BP (20.119 cal B. P.) (UBAR - 935/CNA 089).

Between 2000 and 2003, a maxilla (CF-1), four cranial fragments (CF-2, CF-3, CF-4 and CF-5), a deciduous molar (CF-6) and a fibula (CF-7) were discovered in level 29 Cova Foradà associated to faunal remains and lithic tools. The human remains seem to belong to two individuals. The morphological traits of the human remains, the typology of lithic tools and faunal remains associated to them indicate a Mousterian origin for this layer. The maxilla, the cranial fragments and the shaft of fibula belonged to an adult individual and the deciduous lower right second molar belonged to a 2.5 years child. The specimen described in this study corresponds to a nearly complete maxilla (CF-1) bearing a few teeth [6].

## Materials and Methods

No permits were required for the described study, which complied with all relevant regulations. The current regulation is this: Law 9/1993, 30th of setember, Catalan cultural heritage (DOGC number 1807, 11.10.1993).

**Figure 2:**
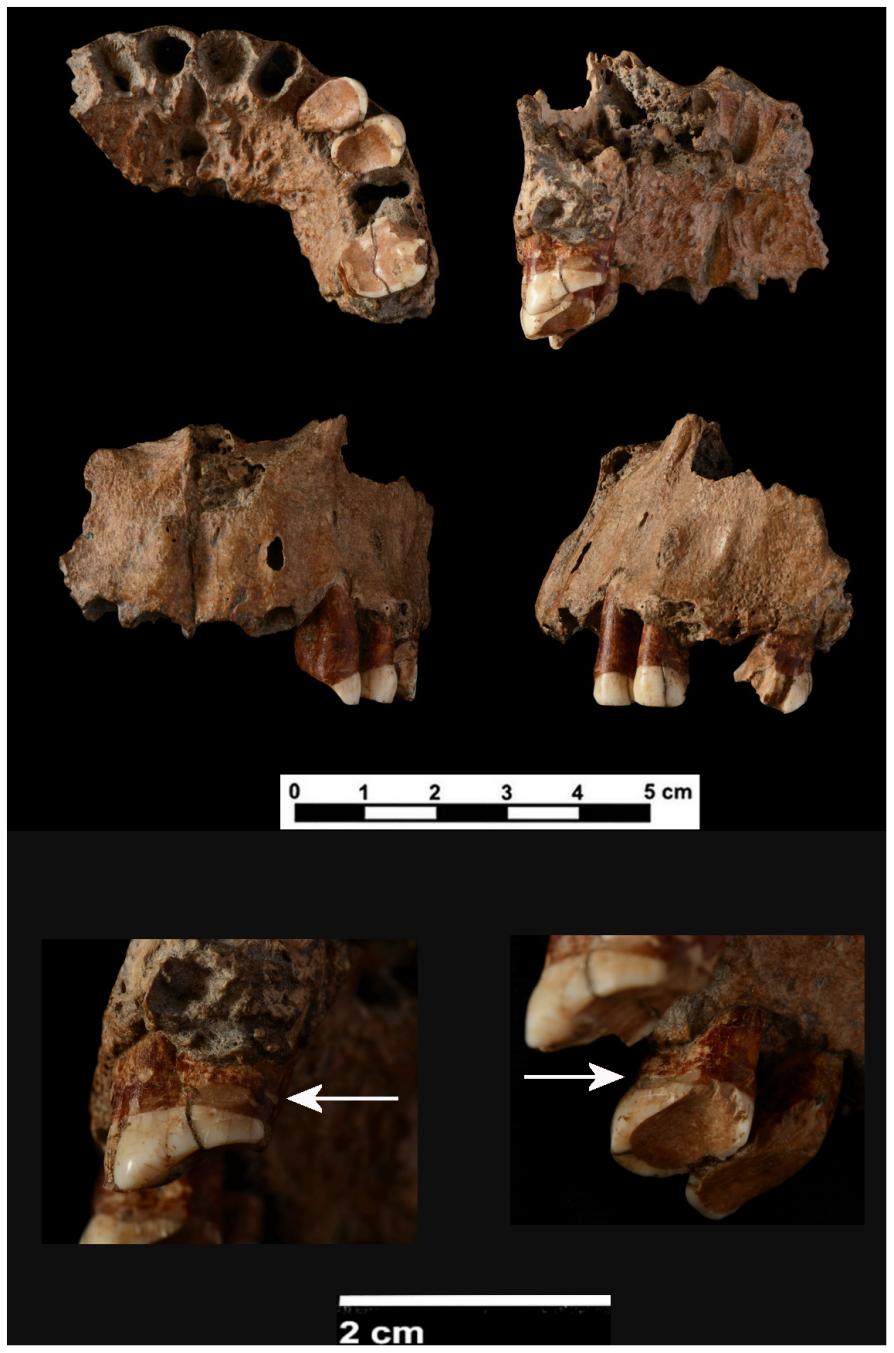
Different general views (inferior, internal, frontal and left) of the maxilla from Cova Foradà site. Detailed images of the last row: lower left, the arrow shows the interproximal groove on distal surface of left M^1^. Lower right, the arrow shows the interproximal groove on distal surface of left Pm^3^.

The material studied consists of three teeth present *in situ* in the articulated right and left adult maxilla, labelled CF-1 which is temporary housed at Institut Català de Paleoecologia Humana i Evolució Social (IPHES) in Tarragona, Spain (Fig. 2). The maxilla comprises the alveoli from the right canine to the second left molar. It contains the left part of the inferior nasal border, the anterior nasal spine and the nasoalveolar clivus. Three teeth remain in place: the left C, Pm^3^ and M^1^. The lack of signs of new bone formation in the alveoli indicates that the other teeth were lost postmortem. The interproximal wear facet on the distal face of the left M^1^ indicates that the M^2^ was erupted. The dental wear analysis suggests an estimated age at death of 35–45 years for this individual [6].

Both maxilla and dentition were analysed and examined for oral pathologies such as periodontal disease, dental caries, dental calculus and abscesses. We scored the presence/absence of alveolar resorption on the CF-1 maxilla. Alveolar resorption is often related to periodontal disease and the progressive degeneration of dental support tissues [7], [8].

The focal demineralization of hard dental tissues is a chronic process caused by bacteria attached to dental plaque and commonly known as dental caries [9]. In archaeological remains its presence/absence in each tooth is documented along with its location (occlusal, coronal, enamel joint and/or root) and the degree of destruction of dental tissues (enamel only, dentine and/or pulp cavity) [10].

We also recorded the presence/absence of dental calculus or calcified dental plaque adhered to dental surfaces and indicated whether it was of the subgingival or supragingival type [11]. Finally, we looked for evidence of periapical abscesses in the maxilla associated with dental decay or other possible oral infections. The maxilla was scanned using computed tomography (CT) with a Toshiba Aquilon CT scanner at the Hospital General de Catalunya in Sant Cugat (Barcelona), using the following scan parameters 120 Kv, 150 mAs, a slice thickness of 1.0 mm, slice increment of 0.3 mm and a pixel resolution of 0.137 mm.

The preserved teeth show heavy dental wear. The crowns were worn down to almost the level of the cementoenamel junction. Dental wear stages were determined in accordance with two different methods, one for anterior teeth and one for posterior teeth. Canine wear was established in keeping with Skinner’s model for anterior teeth [12]. The dental wear of posterior teeth was determined using Murphy’s pattern, as modified by Smith [13]. Individual age at death was estimated using Brothwell’s pattern of dental wear [11].

The heavy wear on the dental surfaces was analysed by means of observation using a Fei Quanta 600 environmental scanning electron microscope (ESEM). High-resolution replicas of the maxillary teeth were made in order to improve inspection in the ESEM chamber [14], [15]. Interproximal grooves were morphologically described and measured with ESEM digital images captured at different magnifications (between ×30 and ×500), 15 kv voltage and a variable working distance of between 20 and 30 mm.

## Results

The three preserved maxillary teeth are characterised by heavy dental wear (Fig. 2). The canine shows stage eight wear on Skinner’s scale for anterior teeth [12]. Only the cervical third of the crown is preserved on the buccal surface. The occlusal wear is tilted toward the tongue and the palatine surface of the tooth is completely depleted; the exposed root is also worn. The occlusal surface shows the formation of secondary dentine and only a thin enamel rim is preserved near the vestibular surface. The Pm^3^ and M^1^ show heavy wear corresponding to stage seven on Murphy’s scale [13]. Enamel in the cervical third of the premolar crown has been preserved on the buccal surface. However, the palatine surface is worn, tilted from the mesial to distal and diminished to the cervicoenamel junction on the distal side. On the central part of the occlusal surface, the dentine has worn to the point that it is concave. The occlusal surface of the M^1^ shows secondary dentine exposed with oblique wear reaching the cervical region on the mesial side. An enamel rim has been preserved on the distal side and there are small spots of enamel on the mesial side and on the buccodistal cusp. The buccal surface of the M^1^ features a large break with enamel and dentine loss also affecting the root. This break could be of antemortem origin, but was enlarged postmortem. The occlusal surfaces of the teeth were examined by means of ESEM. The exposed dentine appears shiny and polished with a few pitted and scratched areas. Some polished and worn chips have been documented on the enamel rim surrounding the occlusal dentine.

There is no evidence of dental caries, dental calculus, abscesses or bone perforation related to a cyst in the Cova Foradà dentition after visual inspection of the maxilla and the analysis of CT images. However, periodontal disease has been documented on the left side of the maxilla [16]. The porous appearance of the alveolar margin and alveolar bone resorption between Pm^3^ and M^1^ could be related to periodontal disease [7]. Alveolar resorption is usually associated with periodontal disease and the degeneration of the soft tissues supporting the teeth [7]. Another useful indicator of periodontal disease is the deterioration of the alveolar bone. The lack of three to six millimetres of alveolar bone is considered mild to moderate periodontal disease, whereas the loss of six or more millimetres denotes serious periodontal disease [8]. The distance between the cervicoenamel junction and the alveolar margin is 8.05 mm on the canine, 4.65 mm on the Pm^3^ and 3.8 mm on the M^1^. The CF individual was therefore affected by mild to serious periodontal disease.

**Figure 3:**
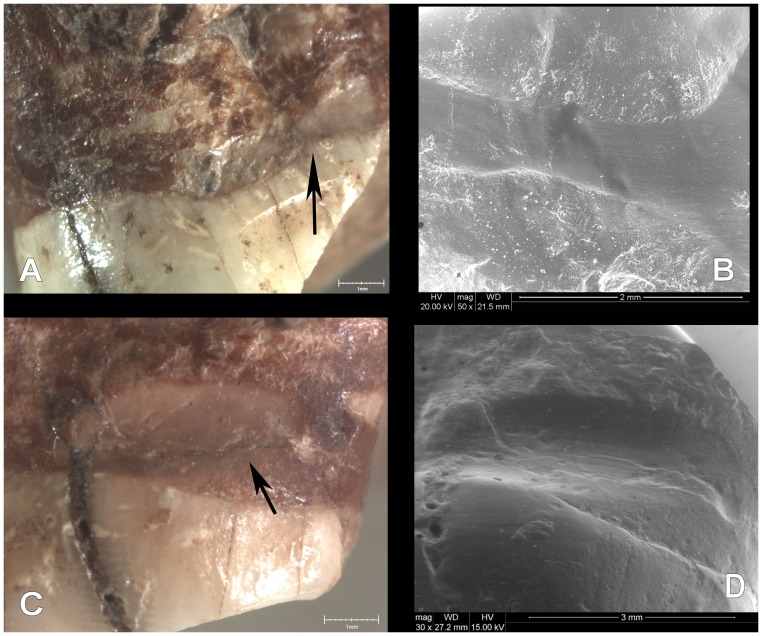
A: Interproximal groove on distal surface of left Pm^3^ (binocular lens image). B: Detailed view of the interproximal groove on Pm^3^ (ESEM image, 100×). C: Interproximal groove on distal surface of left M^1^ (binocular lens image). D. Detailed view of the interproximal groove on M^1^ (ESEM image, 30×).

Two interproximal grooves were found on the distal surfaces of the Pm^3^ and the M^1^. In both cases, the bucolingually elongated grooves are located under the cervicoenamel junction and confirm the presence of periodontal disease. The Pm^3^ groove runs along the entire distal surface, but is more defined and deeper in the half closer to the tongue. After this, the groove splits into two halves (Fig. 3). The groove is 1 mm wide and 5.66 mm long. The M^1^ groove is located on the lingual side of distal surface and measures 4.69 mm in length and 1.1 mm in width. Both grooves have semicircular cross-sections with worn and softened walls, indicating antemortem formation. The ESEM examination shows parallel microscratches bucolingually oriented at the bottom of the grooves (Fig. 3). The other surfaces of the teeth do not show any evidence of interproximal grooves.

## Discussion and Conclusions

The CF-1 maxilla shows evidence of periodontal disease and heavy dental wear, although it lacks other pathologies such as dental decay or abscesses. The heavily worn teeth in this specimen are evidenced by the loss of the majority of the dental crown and, on the occlusal surface, by large areas of exposed dentine surrounded by a thin rim of remnant enamel. The occlusal surfaces show pitting, scratching and enamel chipping of antemortem origin, indicating the intense use of the teeth. The extreme and heavy occlusal load on the teeth throughout the lifetime of this individual resulted in this type of dental wear [17]. Highly abrasive diets containing various hard items demand heavy occlusal loads. A previous study on the dental microwear of the teeth of CF-1 concluded that the vestibular surface of the upper M1 also shows evidence of a highly abrasive diet [16].

The alveolar resorption in CF-1 is severe in the canine alveolus and mild in the other teeth. The periodontal disease of this individual could be considered mild to serious with a generalised gum inflammation that reached the bone. Poor oral hygiene and the long-term effects of plaque deposition can cause gingivitis that degenerates into periodontal disease without treatment.

Interproximal grooves were documented on the distal surfaces of the Pm^3^ and M^1^. They were caused by the action of pulling some type of hard, narrow object, such as a toothpick, between adjacent teeth. Both interproximal grooves are related to heavy dental wear and periodontal disease. The adjacent teeth, the left Pm^4^ and upper left M^2^, were lost postmortem because there is no evidence of new bone formation in either alveoli. We do not know if those teeth suffered any dental pathology such as dental decay. However, the compensatory eruption of teeth with root exposition as a consequence of heavy dental wear could facilitate the introduction of food detritus between the teeth causing irritation and pain. The use of a small piece of wood or bone as a toothpick would alleviate that discomfort. The habit of picking the teeth causes interproximal grooves such as those found in an experimental study using grass stalks [18].

Interproximal grooves have been widely documented on the teeth of fossil populations (Table 1). A lower molar of *Homo erectus* (OH60) from Olduvai dated to 1.84 m.a. and the upper premolar of L894-I from Omo dated to 1.8 m.a. present the earliest evidence of these grooves [4], [18]. This feature is broadly documented in other hominins such as *Homo heidelbergensis* from the Sima de los Huesos site (Sierra de Atapuerca, Spain) and in Neanderthals [1], [19], [20], [21], [22], [23], [24] (Table 1). In our own species, *Homo sapiens*, interproximal grooves have been documented from Upper Palaeolithic individuals to present day aboriginal populations [20], [25], [26], [27], [28] (Table 1).

**Table 1:** Populations with interproximal grooves on teeth.

Site	Species	Teeth affected	Surface	Width	Length
L894-I Omo	*Homo habilis*	Upper Pm3	Distal	n.d	n.d
OH60 Olduvai	*Homo erectus*	Lower M3	Mesial	2.1	6.1
Zhoukoudian	*Homo erectus*	Upper and lower molars	Mesial	n.d	n.d
Sima de los Huesos (Sierra de Atapuerca)	*Homo heidelbergensis*	Upper and lower M1, M2	Mesial/Distal	n.d	n.d
**Cova Foradà**	**Neanderthal**	**Upper M^1^**	**Distal**	**1.1**	**4.69**
		**Upper Pm^3^**	**Distal**	**1**	**5.66**
Hortus IX, XI	Neanderthal	Molars	Mesial/Distal	n.d	n.d
La Quina V	Neanderthal	Lower M_1_, M_2_	Mesial/Distal	0.04–0.05	n.d
La Ferrassie II	Neanderthal	Upper M^2^	Distal	n.d	n.d
La Chapelle-aux-Saints	Neanderthal	Upper M^3^	Mesial	n.d	n.d
Stajnia Cave	Neanderthal	Upper M^2^	Mesial	n.d	n.d
Banyoles mandible	Neanderthal	Lower M_2_	Distal	4	n.d
Krapina	Neanderthal	Incisor, premolars and molars	Mesial/Distal	n.d	n.d
Gibraltar I	Neanderthal	Upper molars	Distal	n.d	n.d
Grimaldi Caves (Barma Grande 5,Grotte des Enfants 4)	*Homo sapiens*	Incisors and molars	Mesial/Distal	0.5–3.5	n.d
Mesolithic populations of Taforalt, Afalou-bou-Rhummel, Mechta-Chceaudun (North Africa)	*Homo sapiens*	Upper and lower molars	Mesial/Distal	1–3	n.d
Lauricocha (Perú) 9525 BP	*Homo sapiens*	Upper M^2^, M^3^	Mesial/Distal	3.3	7.5
Neolithic population of Gotland (Sweden)	*Homo sapiens*	Incisors and premolars	Mesial/Distal	n.d	n.d
Redeyef (Tunis), Neolithic	*Homo sapiens*	Upper molar	Distal	n.d	n.d
Peyraoutes (France), Bronze Age	*Homo sapiens*	Incisor, Upper molar	Distal	n.d	n.d
Puig Anserich (Spain) Bronze Age	*Homo sapiens*	Upper canine	Mesial	3	n.d
Prehistoric populations of Canary Islands	*Homo sapiens*	Upper and lower molars	Mesial/Distal	n.d	n.d
Prehistoric Indians from California	*Homo sapiens*	Mandibular anterior teeth	n.d	1.5–2.2	n.d
Arikara Indians	*Homo sapiens*	Molars	Mesial/Distal	2	n.d
Australian Aborigines	*Homo sapiens*	Molars	Distal	n.d	n.d

All measures are in mm. “n.d” mean no data available for this measure. Data obtained from [1], [4], [18], [19], [20], [21], [22], [23], [24], [25], [26], [27], [28], [30], [31], [32], [33].

In all populations and individuals, groove morphology is characterised by a tubular cross-section and labiolingual orientation. Interproximal grooving is more common on molars and premolars than on anterior teeth. There is no clear preference for mesial or distal surfaces, and there are many examples with both surfaces affected (Table 1). It is always caused by the introduction of some type of hard, thin and rigid probe between the teeth.

Interproximal grooves on healthy teeth can be caused by using a pick between the teeth in order to remove food particles [2], [3], [22], [29], [30], [31], [32], [33]. Another aetiology for grooving on anterior and posterior teeth is linked to the use of the teeth as tools [27]. However, when grooves are documented on teeth with dental decay, heavy dental wear and periodontal disease, they could be the result of attempts to mitigate the inflammation of the gums [26], [28]. The interproximal grooves in CF-1 may be related to this last aetiology because both grooves are associated with periodontal disease and severe dental wear.

In the last years, the evidence of the complex cognitive and behavioural capabilities of the Neanderthals has been increased. It has been proved that Neanderthals could speak and they were right-handed in the same proportion of modern people [34], [35], [36]. Neanderthals had a complex cultural organisation with an important symbolic behaviour such as burials or the use of feathers and claws as a personal garment demonstrate [37], [38], [39]. They also had thorough knowledge of the natural resources of their environment. Neanderthals from El Sidrón Cave (Spain) developed the ability to use medicinal plants, so they had some knowledge of medical treatment [40]. The use of toothpicks of plant origin to mitigate sore gums could also be considered as a type of rudimentary dental treatment.

In sum, the use of toothpicks can be considered one of the most ancient habits documented in our genus, *Homo*. Sometimes, this habit may be related to a primitive form of oral hygiene to remove food particles. But, if interproximal grooves are associated with a dental pathology such as that suffered by the Cova Foradà specimen, the habit of using a tool to pick the teeth may be considered early evidence of medical treatment to alleviate sore gums.
